# Curcumin alleviates imiquimod‐induced psoriasis‐like inflammation and regulates gut microbiota of mice

**DOI:** 10.1002/iid3.967

**Published:** 2023-08-09

**Authors:** Zhenguo Cai, Wuqing Wang, Yao Zhang, Yibin Zeng

**Affiliations:** ^1^ Department of Dermatology, Minhang Hospital Fudan University/Central Hospital of Minhang District Shanghai China; ^2^ Department of Dermatology Shuguang Hospital Shanghai University of Traditional Chinese Medicine Shanghai China; ^3^ Dermartment of Dermatology The First Affiliated Hospital of Soochow University Suzhou China

**Keywords:** curcumin, gut microbiota, inflammation, psoriasis

## Abstract

**Background:**

As a polyphenolic compound originated from the food spice turmeric, curcumin (CUR) has various pharmacological effects, such as anti‐inflammatory, antioxidation, antiproliferative, and antiangiogenic activities. Psoriasis is centered on the overproduction of Th1‐ and Th2‐related cytokines (e.g., interleukin [IL]‐23, IL‐17, TNF‐α, IL‐22), which is involved in the occurrence and development of its pathogenesis. However, whether CUR is involved in the treatment of psoriasis and its specific mechanisms are not fully understood.

**Methods:**

In this study, we detected the therapeutic effect of CUR (100 mg/kg/day) on IMQ‐induced dermatitis in mice, analyzed by PASI scores, ELISA, HE staining, immunofluorescence. Moreover, we further confirmed the alteration in the relative abundance of the gut microbiota through 16sRNA to explore whether CUR could regulate the gut microbiota of IMQ‐induced mice.

**Result:**

Through intragastric administration, CUR can alleviate psoriasis‐like lesions of mice by decreasing PASI scores, reducing the level of IL‐6, IL‐17A, IL‐22, IL‐23, TNF‐α, and TGF‐β1, promoting the expression of IL‐10. Moreover, 16sRNA sequencing revealed that CUR could regulate the alteration in the abundance alteration of gut microbiota related to inflammation, such as *Alistipes, Mucispirillum, and Rikenella* at genus level. The correlation analysis further confirmed the close association between important microflora and psoriasis‐like inflammation indicators.

**Conclusions:**

CUR exerts the effect of alleviating dermatitis of psoriatic mice by regulating Th‐17 related inflammatory factors. Moreover, the changes in gut microbiota via CUR may be another factor of relieving IMQ‐induced lesions in mice. Therefore, CUR may be a highly promising candidate for the treatment of psoriasis.

## 1 INTRODUCTION

Psoriasis is a chronic, immune‐mediated skin disease that can be accompanied by joint involvement. For its high prevalence and the complexity of causative mechanisms, psoriasis has caused great concern worldwide.^1^ Its typical clinical manifestations are scales, erythema, and epidermal thickening. Currently, it is well recognized that psoriasis results from the complex interaction between keratinocytes and immune cells.^2^ Many inflammatory factors (e.g., TNF‐α, interleukin [IL]‐1 β, IL‐23, and IL‐12) secreted by Th17 cells, Th1 cells, and keratinocytes played crucial effects in their pathogenesis.^3^ A growing body of evidence suggests that psoriasis is closely linked to multiple comorbidities, such as systemic inflammation and cardiovascular comorbidity.^4^ Therefore, psoriasis has gained significant attention worldwide. However, there are no effective measures to achieve a cure for psoriasis.

Curcumin is a polyphenolic compound purified and isolated from *Curcuma longa*. Since ancient times, turmeric had been considered an herbal remedy for the treatment of skin and gastrointestinal disorders.^5^ Now, it is clear that its widespread use in medicine stems from its many properties, such as antioxidant, anti‐inflammatory, antiproliferative, anticancer, and antibacterial effects.^6^ It had been demonstrated that curcumin can reduce various inflammatory factors such as TNF‐α, IFN‐γ, IL‐22, and IL‐23 in mouse serum, which are closely involved in the pathogenic mechanism of psoriasis.^7^ Meanwhile, curcumin was also considered as a protective compound that can regulate the intestinal microflora. Currently, increasing evidence had found that gut microbes were potentially involved in regulating the progression of a variety of diseases closely related to inflammation, such as psoriasis, psoriatic arthritis, inflammatory bowel diseases (IBD).^8^, ^9^

To further confirm our speculation, we found that curcumin could significantly improve the IMQ‐induced mouse model, and reduce the expression of multiple pathogenic factors in its skin lesions, such as TNF‐α, IL‐17, IL‐1 β, and so on. Furthermore, we analyzed the changes of intestinal microbiota in mice after curcumin treatment. By using 16s rRNA technology, this study investigated whether curcumin could exert its therapeutic effect in psoriasis, which was in relation to the regulation of intestinal flora.

## 2 MATERIALS AND METHODS

### 2.1 Materials

Imiquimod cream (5%) was gained from Sichuan Mingxin Pharmaceutical Corporate, methotrexate was obtained from Chromabio. Anti‐PCNA mouse mAb were obtained from Proteintech. All ELISA kits used in this experiment were purchased from Beyotime.

### 2.2 Experimental sketch

BALB/c mice (8–10 weeks, 20–22 g) used in this study were gained from SPF (Beijing) Biotechnology Co., Ltd. All animal experiments were in accordance with the relevant provisions and obtained the approvement from The Animal Experiment Committee of Fudan University. The mice were shaved a smooth area of 2 × 3 cm at the back. Meanwhile, every mouse could acquire enough food and water during experiment. Their living environment was also dry and comfortable. We randomly divided the mice into four groups: (1) control (CON, *n* = 4); (2) imiquimod (IMQ, *n* = 4) treated; (3) methotrexate (MTX, a clinical drug to treat psoriasis, *n* = 4,) treated; (4) curcumin‐treated group (CUR, *n* = 4). Except the CON group mice were applied topically with the same dose of vaseline cream, the shaved area of other groups mice was applied topically with a daily dose of 62.5 mg imiquimod cream every day. The psoriasis‐like model was built after continuous 6 days. MTX‐treated mice and CUR‐treated mice were treated for the intragastric application with MTX (1.0 mg/kg/day) and curcumin (100 mg/kg/day), applied after 1 day of imiquimod treatment. MTX and CUR were dissolved and diluted by clean and distilled water. After treating the mice with an overdose of the anesthetic drugs, the back skin and intestinal feces were collected for further processing.

### 2.3 Hematoxylin/eosin (HE) staining and immunofluorescence

The acquired mice skin tissues were fixed in 4% paraformaldehyde solution, and paraffin blocks were treated and stained by HE. For immunofluorescence staining (IFC), main steps were performed as described previously.^10^ In addition, the fluorescence intensity of PCNA was calculated by Image J. All sections were examined with laser confocal microsopes (AOSVI, M303‐HK830).

### 2.4 ELISA detection of skin tissues inflammatory factors

After adding the lysate, each collected skin tissue was broken through a crusher to form a solution. The levels of IL‐17, TNF‐α, IL‐1β in mice skin tissues were assessed by ELISA kits (SAIPEISEN BIOLOGY). The main steps followed the manufacturer's instructions.

### 2.5 DNA extraction and polymerase chain reaction (PCR) amplification

Total DNA was extracted from mouse feces using the PowerSoil® DNA Isolation kit according to the manufacturer's instructions. After extraction, the 16S full‐length primer was designed according to the conservative region 27F‐1492R, for polymerase chain reaction (PCR) amplification of the target area (10 μL system, Solexa PCR). The synthesized specific primers (27F‐1492R) were designed as follows: Forward primer 27F: AGRGTTTGATYNTGGCTCAG; Reverse primer 1492 R: TASGGHTACCTTGTTASGACTT.

### 2.6 Sequencing and data analysis

Quality inspection was performed on the formed sequencing library, and processing, including barcode recognition, was performed on the high‐quality circular consensus sequencing (CCS) sequence obtained. The generated optimization CCS was clustered at the level of 97% similarity (USEARCH, version 10.0), and its species classification was obtained based on the sequence composition of the operational taxonomic unit (OTU). The platforms 16S: Silva database and RDP Classifier were used to analyze species annotation and taxonomy as well as the diversity of gut microbiota.

### 2.7 Statistical analysis

All experiment data in this study were performed in triplicate, and statistical analyses were processed with the GraphPad Prism 7.0, which has been showed as the mean ± SD. Statistical significance of all data was examined by one‐way analysis of variance (ANOVA) or Student's *t*‐test as described previously.^11^ A value of *p* < 0.05 was considered statistically significant.

## 3 RESULTS

### 3.1 Curcumin inhibits psoriasis‐like skin lesions induced by IMQ

**Figure 1:**
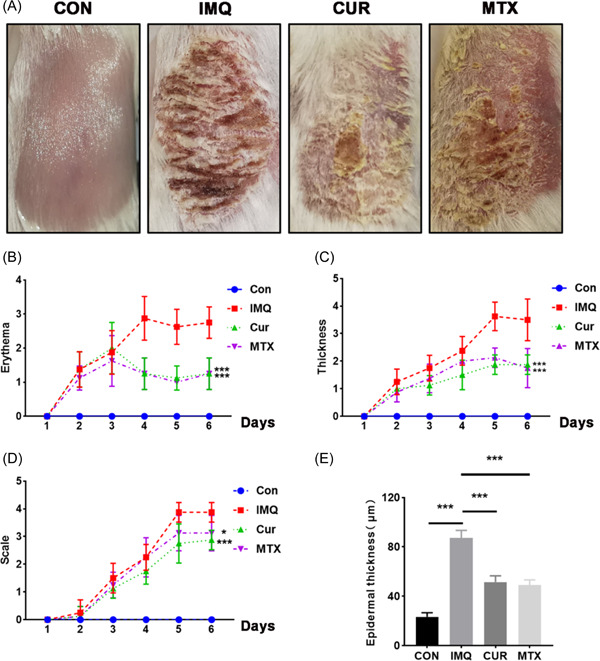
Curcumin (CUR) can ameliorate the lesions of imiquimod (IMQ)‐induced mice. (A) Comparison of back skin lesions on Day 6 of mice in different groups (*n* = 4). (B–D) The psoriasis area severity index (PASI) score of affected skins containing erythema, scale, thickness (*n* = 4). (D) Statistics of back epidermis thickness in mice of different groups (*n* = 4). *p < 0.05, ***p < 0.001.

To further clarify the regulatory effect of CUR on psoriasis, we constructed psoriasis‐like skin lesions induced by IMQ (Figure 1A). As shown in Figure 1B–D, PASI scores were largely increased in IMQ‐induced mice than in control mice. CUR could significantly decrease PASI scores and epidermal thickness in affected lesions of IMQ‐induced mice (Figure 1B–E). Therefore, CUR could relieve the IMQ‐induced mouse skin lesions and reduce the PASI scores in the affected areas.

### 3.2 CUR ameliorate the morphology changes of psoriasis‐like dermatitis in mice

**Figure 2:**
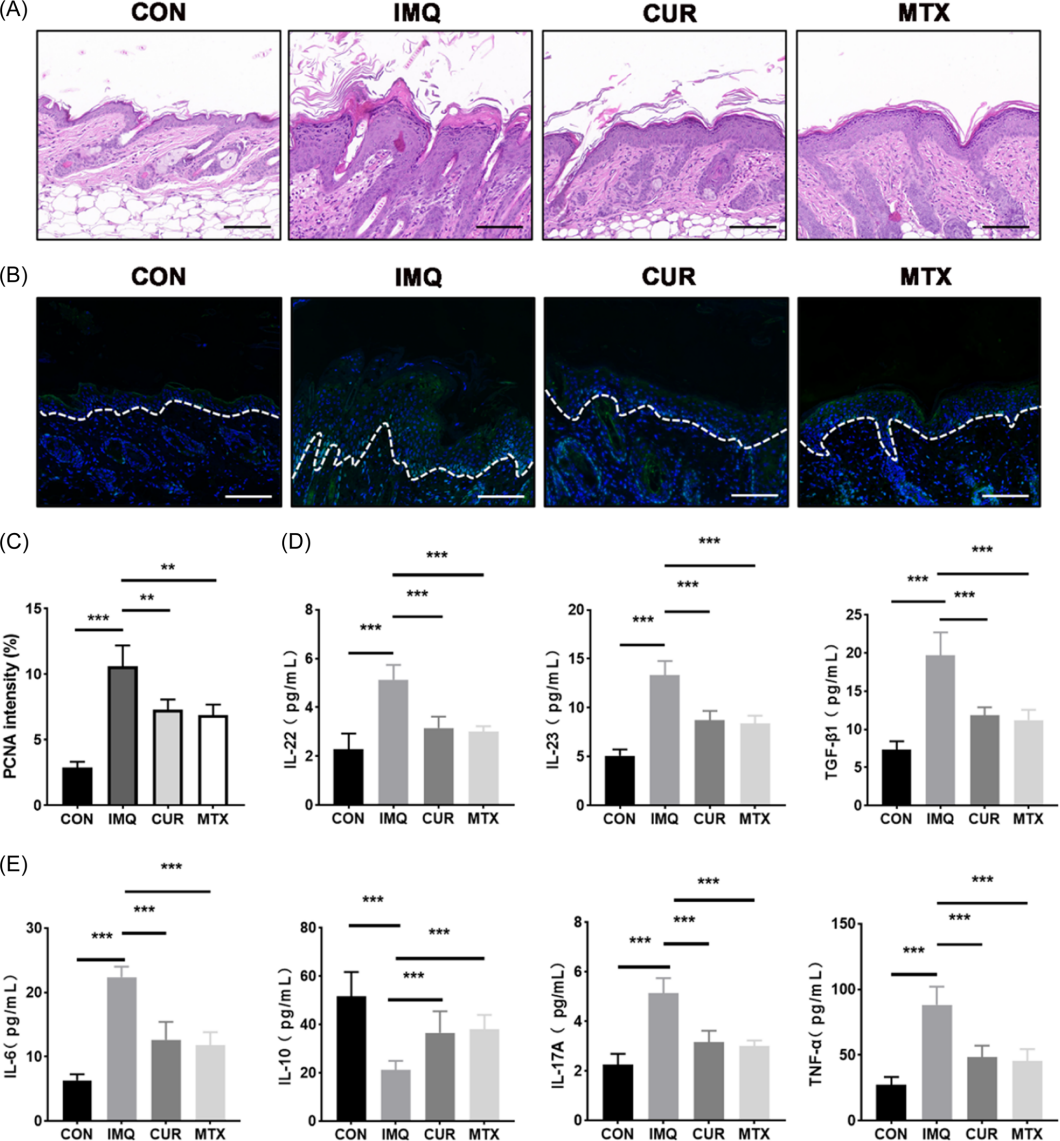
Imiquimod (IMQ) can suppress pathological changes and inflammatory indicators in psoriasis mice. (A) Results of back hematoxylin/eosin (HE) staining of mice after different treatments. Bar = 100 μm. (B) Immunofluorescence staining of PCNA from mouse dorsal skin of different groups. Bar = 100 μm. (C) The fluorescence intensity of PCNA was calculated by Image J. (D, E) Levels of different inflammatory indicators in mouse back skin lesions as assessed by ELISA (*n* = 4). **p < 0.01, ***p < 0.001.

We further processed the skin tissues collected from all groups of mice for HE staining and IFC staining. The results showed that oral curcumin alleviated IMQ‐induced dermatitis in mice, such as parakeratosis, erythema, and infiltrating inflammatory cells (Figure 2A). Moreover, PCNA was a highly proliferative marker of keratinocytes at psoriatic lesions.^12^ The IFC results showed that CUR significantly decreased the expression of PCNA in the back skin of model mice (Figure 2B,C). Thus, the data revealed that CUR can markedly inhibit over‐proliferating keratinocytes in IMQ‐induced lesions of mice.

### 3.3 CUR regulates the inflammatory indicators associated with psoriasis‐like lesions in mice

To further explore whether CUR regulates the inflammatory responses induced by IMQ, we explored the expression levels of multiple inflammatory indicators in mouse skin lesions by ELISA kit. As shown in Figure 2D,E, CUR can significantly the epidermal expression levels of IL‐6, IL‐17A, IL‐22, IL‐23, TNF‐α, and TGF‐β1 compared with IMQ group. In addition, the expression levels of IL‐10 in CUR group were higher than in the IMQ group (Figure 2E). The results demonstrated that CUR can repress the expression of inflammation factors related to psoriasis onset and development.

### 3.4 CUR reshapes the intestinal microflora composition of mice

**Figure 3:**
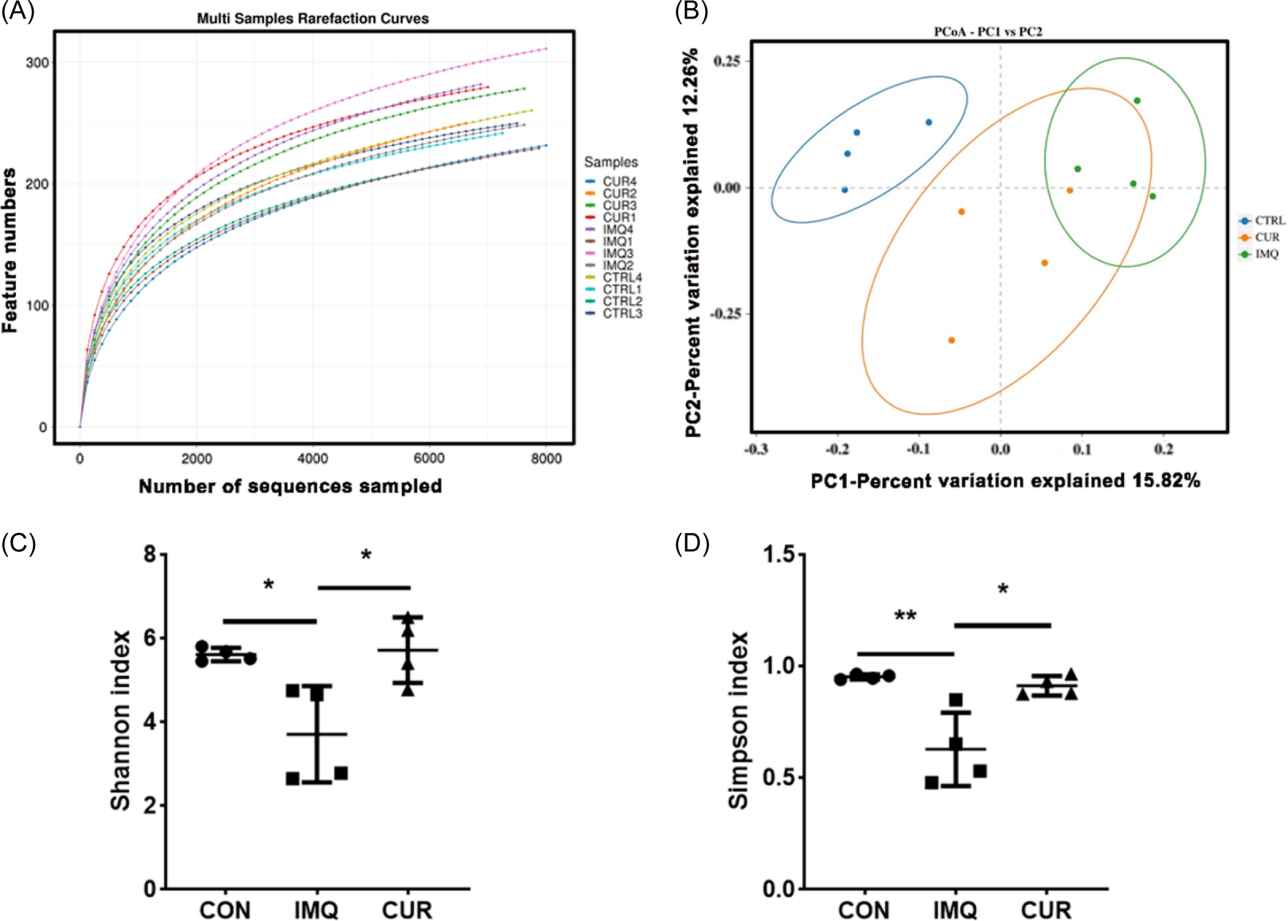
Analysis of the gut microbiota communities between control (CON)‐, curcumin (CUR)‐, imiquimod (IMQ)‐treated groups. (A) Comparison of rarefaction curves in V3‐V4 pyrosequencing tags of the 16sRNA gene in gut microbiota (*n* = 4). (B) principal coordinate analysis (PCoA) comparison between CON‐, IMQ‐, CUR‐ groups. (C, D) α diversity analysis of mice in different groups (*n* = 4). *p < 0.05, **p < 0.01.

**Figure 4:**
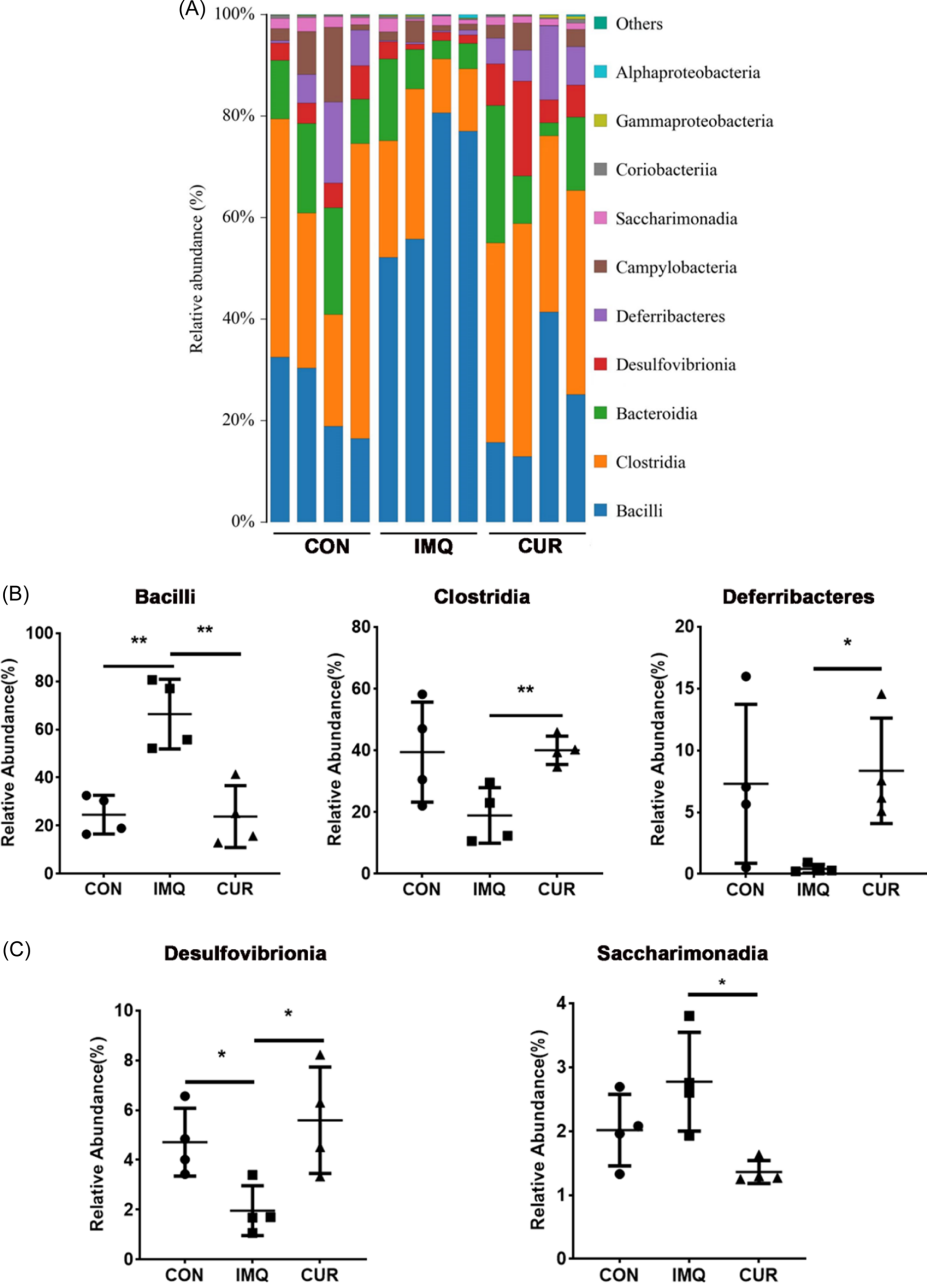
Differences in gut flora between different groups. (A) Relative abundance at the class level (*n* = 4). (B, C) Changes of gut microbes at class level between control (CON)‐, imiquimod (IMQ)‐, curcumin (CUR)‐treated groups. *p < 0.05, **p < 0.01.

Next, we further investigated the effect of CUR on the intestinal microbiota of mice. We examined the V3 and V4 regions within the 16sRNA of all mouse gut microbiota, and the rarefaction curves showed extremely high coverage in every sample (Figure 3A). From the principal coordinate analysis (PCoA) results, we found a significant separation of the microflora distribution between groups CON, IMQ, and CUR (Figure 3B). After analyzing the flora diversity among the CON, IMQ, and CUR groups, Simpson and Shannon index showed that the CON and CUR groups were significantly higher than the IMQ group (Figure 3C,D). Moreover, the analysis of the microflora diversity results showed that it mainly included 10 classes: *Bacilli*, *Clostridia*, *Bacteroidia*, *Desulfovibrionia*, *Deferribacteria*, *Campylobacteria*, *Saccharimonadia*, *Coriobacteriia*, *Gammaproteobacteria*, *Alphaproteobacteria* (Figure 4A).

For further exploring these data, we performed high dimensional analysis by line discriminant analysis effect size (LEfSe) that determined significant differences in the main flora between CON, IMQ, and CUR groups. The dominant flora was Lactobacillus at the genus level IMQ group, which we speculated is due to reduced diversity of microflora. The CUR group showed the dominant flora were *Mucispirillum*、*unclassified_desulfovibrionaceae* at the genus level, while *Deferribacters*, *Desulfovibrionia* at the class level. The CON group revealed that the predominant flora was *Ruminococcaceae* at the family level (Supporting Information: Figure S1A,B).

**Figure 5:**
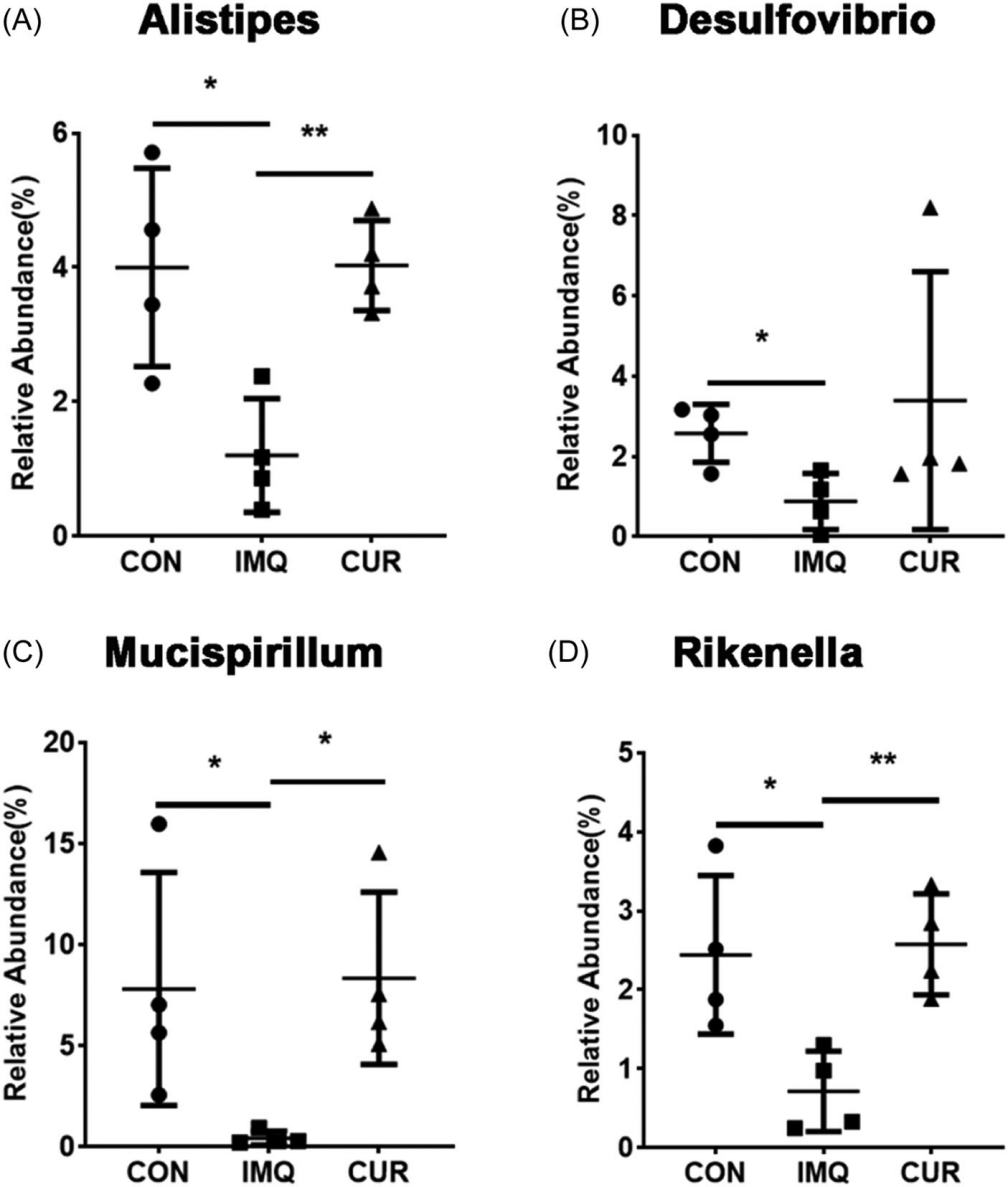
Changes at genus level between control (CON)‐, imiquimod (IMQ)‐, curcumin (CUR)‐treated groups. *p < 0.05, **p < 0.01.

Subsequently, we performed Kruskal–Wallis test to detect the microflora differences between the three groups. Compared with CON and CUR groups, the relative abundance of *Bacilli* at the class level was marked increased in IMQ group, while the result of *Desulfovibrionia* was contrary (Figure 4B,C). Moreover, compared with IMQ group, the relative abundances of *clostridia*, *Deferribacteres*w, and *Desulfovibrionia* at the class level were significantly promoted in the CUR group, while the result of *Bacilli* and *Saccharimonadia* was opposite (Figure 4B,C). Ultimately, at the genus level, the abundance of *Alistipes*, *Desulfovibrio*, *Mucispirillum*, and *Rikenella* in CON and CUR groups was markedly higher than in the IMQ group (Figure 5A–D).

### 3.5 Changed intestinal microflora by CUR was associated with psoriasis‐related factors

**Figure 6:**
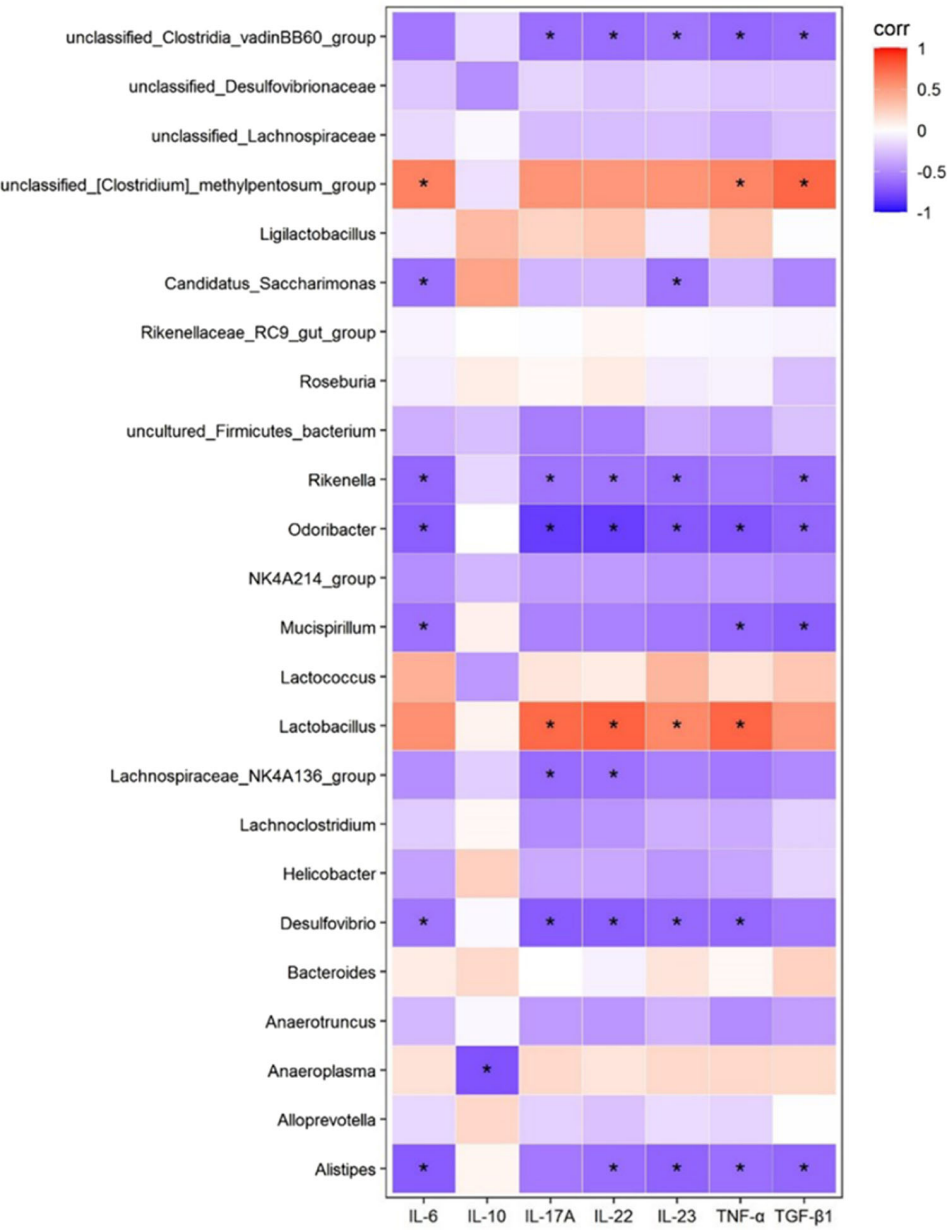
(A) Spearman correlation analysis. The colors ranged from red (positive correlation) blue (negative correlation). *p < 0.05.

To further understand the role of altered gut microbiota by CUR and its connections with psoriasis, we treated psoriasis‐related inflammation indicators as a variable by Spearman correlation analysis at the genus level based on OTU abundance, presented with a heatmap. As shown in Figure 6A, *Ligilactobacillus* and *Anaeroplasma* (inhibited in CUR mice) were positively correlated with multiple psoriasis‐related factors, such as IL‐6, IL‐17A, IL‐22, IL‐23, while *Rikenella, Alistipes*, and *Mucispirillum* (promoted in CUR mice) were negatively correlated with them. These results indicated that the gut flora changes induced by CUR are associated with the inflammatory factors in psoriasis.

## 4 DISCUSSION

Psoriasis is well recognized as a chronic inflammatory disease prone to recurrent attacks. Its typical symptoms are well‐demarcated erythema and skin scales accompanied with systemic symptoms.^13^ Curcumin, as a polyphenolic compound stemmed from turmeric, owns multiple pharmacological effects, comprising anti‐inflammatory, antiproliferative, antioxidant, and antiangiogenic activities.^14^

It is well known that the typical histological changes of psoriasis show excessive proliferation of keratinocytes and infiltration of inflammatory cells, increased PCNA in skin lesions, and elevation of various inflammatory factors, such as IL‐6, IL‐17A, IL‐22, IL‐23, IL‐1β.^15^, ^16^ We found that CUR could significantly alleviate IMQ‐induced psoriasis‐like dermatitis, decreasing lesion thickness of mice, inflammatory cell infiltration, dyskeratosis, and spinous layer hypertrophy. In additions, CUR could inhibit the levels of PCNA in the epidermis of IMQ‐induced mice, and the epidermal expression of IL‐6, IL‐17A, IL‐22, IL‐23, TNF‐α, TGF‐β1. Thus, the results revealed a possible effective mechanism of CUR against psoriasis‐like dermatitis induced by IMQ.

Recently, reports had shown that the occurrence and development of psoriasis were closely associated with intestinal microbiota, and some pro‐inflammatory gut microbes promoted Th1/Th17 activation in psoriasis, which then could cause the expression of various inflammatory factors in the epidermis, such as IL‐17 and TNF‐α.^17^ Therefore, the intestinal microbiota can be regarded as a potential therapeutic target for alleviating psoriasis. Meanwhile, it had been shown that CUR can participate in the regulation of intestinal microbiota.^18^ Taken together, we reasoned that CUR reshaped the diversity of the mouse gut microbiota.

To further investigate the supposition, we detected the effect of CUR on intestinal flora by using 16S rRNA sequencing. Remarkably, we found that the abundances of genus *Alistipes*, *Rikenella*, and *Mucispirillum* were promoted in CUR group. The researchers found that *Alistipes* and *Odorbacter* were positively correlated with intestinal acetate and propionate production, which inhibits the inflammatory activity of IBD.^19^ *Rikenella* had been shown to be associated with the formation of short‐chain fatty acids (SCFAs). It had been reported that the intestinal microflora and its metabolites can catabolize polysaccharides to produce SCFAs. SCFAs can maintain the intestinal barrier function, which is important for intestinal homeostasis. SCFAs not only maintained the cellular barrier but also prevented the transfer of LPS from the intestinal barrier.^20^ Moreover, the abundance of *Mucispirillum* positively correlated with the concentration of the inflammatory suppressor IL‐10 in breast cancer model mice.^21^ Interestingly, *Lactobacillus* at the genus level was increased, which was probably due to the decreased diversity of gut microbiota of mice in the IMQ group.

Taken together, although there was no direct evidence to demonstrate that the gut flora changes induced by CUR linked with psoriasis, these bacteria were associated with inflammation. Furthermore, Moreover, the correlation analysis further showed a significant association between the three picked gut microflora at genus level regulated by CUR and inflammatory factors. We thought that there was a close connection between them. Therefore, inflammation served as an important role to connect CUR, psoriasis, and intestinal microbiota together. To be brief, CUR could relief psoriasis‐like lesions of mice by repressing Th‐17‐related inflammatory factors, and regulate intestinal microbiota associated with inflammation, showing CUR may be a promising drug for alleviating psoriasis.

## AUTHOR CONTRIBUTIONS

**Zhenguo Cai**: Conceptualization; data curation; formal analysis; investigation; supervision; validation; writing—original draft; writing—review and editing. **Wuqing Wang**: Conceptualization; data curation; formal analysis; funding acquisition. **Yao Zhang**: Conceptualization; data curation. **Yibin Zeng**: Conceptualization; data curation; formal analysis; funding acquisition; investigation; methodology; project administration; writing—original draft; writing—review and editing.

## CONFLICT OF INTEREST STATEMENT

The authors declare no conflict of interest.

## Supporting information

Supporting information.

## Data Availability

All of the data presented in this article were available.
