# Supplementary material for: Curcumin alleviates imiquimod‐induced psoriasis‐like inflammation and regulates gut microbiota of mice
Source: Immun Inflamm Dis. 2023 Aug 9;11(8):e967. doi: 10.1002/iid3.967 (PMC10411394; doi:10.1002/iid3.967)
Supplement: Supplementary file 1 — Supporting information. [file IID3-11-e967-s001.docx]

**Supplementary Figure. 1. (A)** LDA score assessed from features differentially abundant between CON-, IMQ-, and CUR-treated mice. The inclusion criteria is log LDA score ＞4. **(B)** Taxonomic cladogram gained from linear discriminant analysis effect size analysis of 16S sequences.

**
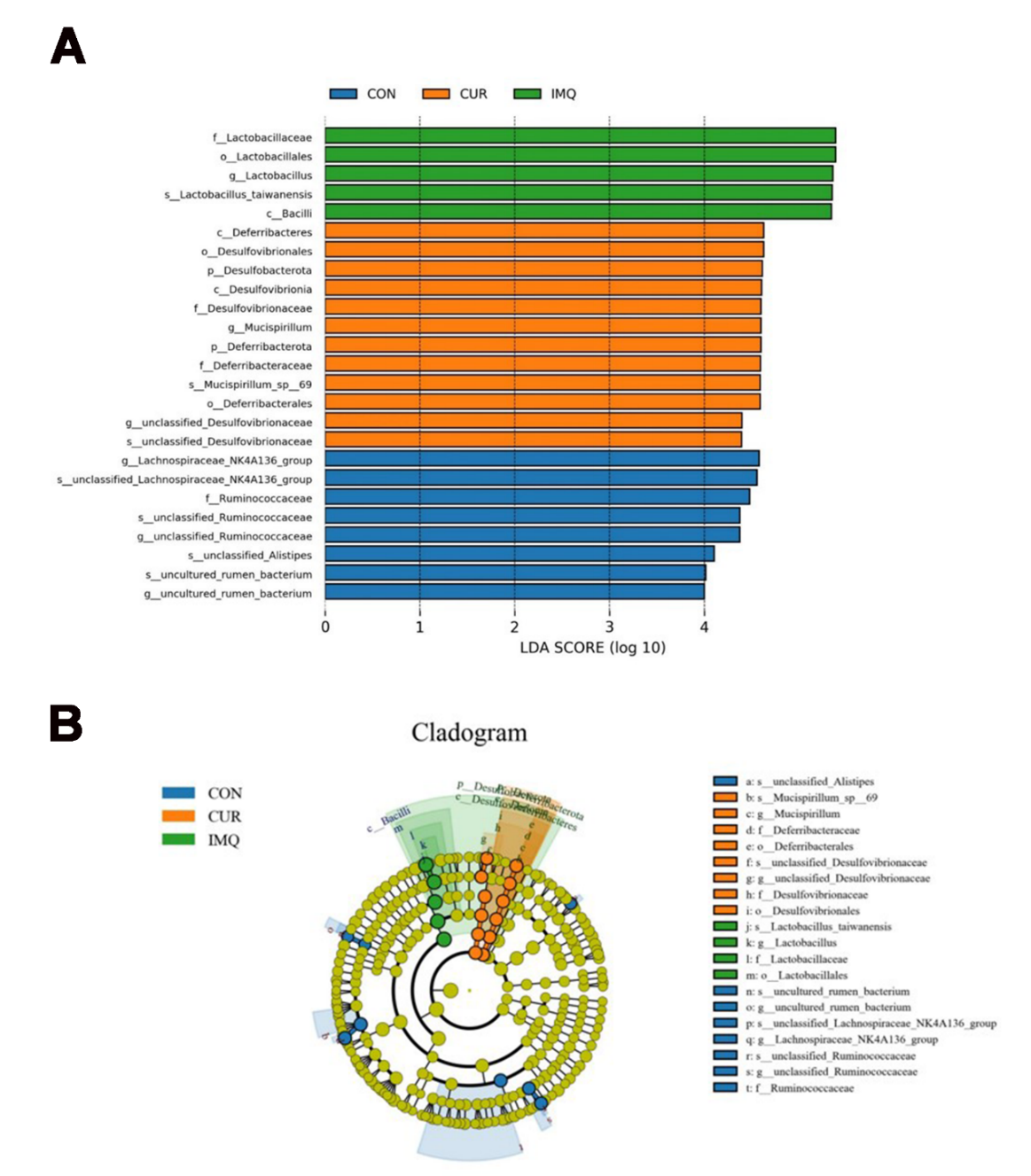
**
